# Atraumatic Limping Child, a Challenge for Pediatricians: An Observational Age-Related Study in a Pediatric Emergency Department

**DOI:** 10.3390/children11020185

**Published:** 2024-02-02

**Authors:** Sebastian Cristaldi, Alessandra Boni, Valentina Ferro, Antonio Musolino, Nicoletta Della Vecchia, Elena Boccuzzi, Elena Bellelli, Francesco Saverio Biagiarelli, Angelo Gabriele Aulisa, Marco Cirillo, Umberto Raucci, Alberto Villani

**Affiliations:** 1 General Pediatrics and ED 2nd Level, Bambino Gesù Children’s Hospital, IRCCS, 00165 Rome, Italy; sebastian.cristaldi@opbg.net (S.C.); valentina.ferro@opbg.net (V.F.); nicoletta.dellavecchia@opbg.net (N.D.V.); elena.boccuzzi@opbg.net (E.B.); elena.bellelli@opbg.net (E.B.); fsaverio.biagiarelli@opbg.net (F.S.B.); alberto.villani@opbg.net (A.V.); 2 Pneumology and Cystic Fibrosis, Bambino Gesù Children’s Hospital, IRCCS, 00165 Rome, Italy; alessandra.boni@opbg.net; 3 Residency School of Pediatrics, University of Rome Tor Vergata, 00133 Rome, Italy; antonio.musolino@opbg.net; 4 Orthopaedics and Traumatology Division, Bambino Gesù Children’s Hospital, IRCCS, 00165 Rome, Italy; agabriele.aulisa@opbg.net; 5 Department of Radiology, Bambino Gesù Children’s Hospital, IRCCS, 00165 Rome, Italy; marco.cirillo@opbg.net; 6 Systems Medicine Department, University of Rome Tor Vergata, 00133 Rome, Italy

**Keywords:** atraumatic, limp, child, emergency, pediatrics

## Abstract

Background: Atraumatic limping is a frequent cause of consultation in Pediatric Emergency Departments (PED) and often represents a challenge for pediatricians for its variability in etiology ranging from benign causes to potential crippling conditions. The aims of this research are to illustrate the clinical features of acute limping children (LC) and to identify the possible red flags that could help to make a diagnosis of severe pathologies. Methods: We carried out a retrospective study about non-traumatic limping children referred to the PED of Bambino Gesù Children’s Hospital over a 2-year period. We divided the cohort into three groups based on the patient’s age: toddlers, children and adolescents. We considered crippling conditions: oncologic etiologies, bone or neurological infections, epiphysiolysis, Perthes disease, Guillain Barrè syndrome and non-accidental injuries. Results: We analyzed 485 patients. At clinical evaluation, 19.5% of the patients presented at least one sign and/or symptom of red flags. Crippling conditions (6.2% of the total population) showed red flags in 36.7%. Transient synovitis of the hip was the most frequent diagnosis. We found crippling conditions in 30 patients, mostly represented by toddlers. Conclusions: Our data suggest that toddlers and patients presenting red flags should be evaluated with particular suspicion because they have an increased risk of underlying severe conditions.

## 1. Introduction

Limping is a frequent cause of consultation in Pediatric Emergency Departments (PEDs) and can represent a challenge in clinical practice for the wide range in nature and severity of the potential underlying etiologies. The exact incidence of atraumatic limping children (LC) is difficult to establish; the few existing data in the literature show an incidence ranging from 1.8 to 2.8 children out of 1000, an average age of 4.4–5.2 years old and a slight male predominance (male-to-female ratio of 1.7:1) [1,2]. 

Limping is defined as a deviation from the normal age-appropriate metric and time parameters of the gait pattern, such as antalgic gait and non-antalgic limp as Trendelenburg gait (the child shifts his or her body weight over the affected hip during the stance phase) [3].

Differential diagnosis in the evaluation of a limping pediatric patient may include benign or self-limiting causes, such as transient synovitis of the hip (TSH), but also serious conditions requiring prompt treatment in order to prevent morbidity and mortality, including malignancies, osteomyelitis, septic arthritis and rheumatologic diseases; in this perspective, clinicians have to avoid potential complications on the one hand and optimize costs, time and investigations on the other.

A proper clinical approach in the diagnostic algorithm must take into account the following: age of the patient, history of recent traumas, number and pattern of involved joints, signs of infection, the timing of onset of symptoms and the presence of “red flags” [4,5]. 

TSH constitutes the most frequent diagnosis made for pediatric patients presenting in PEDs for limping with no history of previous trauma [6]; it mainly affects children aged from 3 to 10 years old, showing, in most cases, neither fever nor other systemic symptoms and with a duration of less than one week [2].

It is often difficult to visit complaining children in the PED, to investigate their clinical history and to assess the need and timing of follow-up; nonetheless, a delay in diagnosis can sometimes result in potential sequelae for the patient, such as in the case of septic arthritis or osteomyelitis [2,4].

Another point of discussion is in regard to the indications and type of imaging within the diagnostic work-up in a PED setting in such cases. Indeed, sometimes serious diseases such as osteomyelitis can present with no radiologic findings within the first days after the onset of symptoms.

Several studies have proposed different algorithms for differential diagnosis and management of limping in pediatric patients, based on either clinical signs, blood markers or imaging findings, but still with no consensus on the best approach and no validated risk prediction tools [5,6,7,8,9,10,11,12,13,14,15] aside from only a few studies focused on the PED setting [4,12,16].

The most common age-related diseases in pediatric patients presenting with atraumatic limp are represented in Table 1 [3,5].

The purposes of our study were to evaluate the epidemiology of limping children in a tertiary care children’s hospital and to estimate the main etiologies based on age; we also tried to assess the validity of red flags and other clinical features or blood markers in order to distinguish self-limited conditions from severe ones in PEDs. 

## 2. Materials and Methods

### 2.1. Data Collection

After obtaining approval from the institutional ethic committee, we conducted a retrospective cohort single-center study about atraumatic limping patients aged from 12 months to 16 years old, presenting to the PED of Bambino Gesu’ Children’s Hospital, a tertiary pediatric hospital, over a 2-year period. In our hospital, a pediatrician is responsible for the initial evaluation, whereas referral to a specialized consultant for a definitive diagnosis is considered the next step for children with an unclear diagnosis.

Patients were identified through keyword searches in the hospital’s electronic databases encompassing all patients who sought care at the PED from 1 January 2010 to 31 December 2019, specifically for acute atraumatic LC. The search criteria were applied to the field’s ‘history’, ‘clinical examination’, and ‘diagnosis’. Subsequently, potential cases were manually selected based on a review of medical records.

In our PED, the prioritization of consultations adhered to a 4-color triage coding scale in accordance with the Italian Health System Guidelines applicable during the study period [17]. For the purposes of this study, PED consultation priorities were categorized as follows:

1. High/Intermediate Priority: This category encompasses patients classified as “code red” (indicating critical medical status) and “code yellow” (indicating a severe status with a risk of evolving into a critical condition).
2. Low/Non-Urgent Priority: This category encompasses patients classified as “code green” (indicating a fair status with stable vital signs) or “code white” (indicating a good status and non-urgent consultation).

Demographic and clinical data were extracted from medical records: age, gender, triage code, time of onset symptoms, history of a previous limp or PED access for the same reason, physical examination findings, comorbidities, presence of “red flags”, values of blood exams, specialist consultations and instrumental investigations requested at a PED, final diagnosis, hospital admission and length of hospitalization, where applicable.

According to the literature, we considered the following as “red flags”: fever, night sweating, night pain, weight loss, fatigue, absence of limb movement, headache and meningeal signs.

We divided our cohort into 3 groups based on patients’ age: toddlers (<3 years), children (3–10 years) and adolescents (>10 years), according to the previous literature [2,4,5]. 

We categorized final diagnoses other than TSH into different groups by subspecialty type: rheumatological diseases, neurological diseases, infectious diseases, oncological, metabolic-nutritional, neuropsychiatric and musculoskeletal diseases.

For hospitalized patients, we considered the diagnosis made at the time of discharge from the hospital as our final diagnosis.

We identified the following as “crippling/severe conditions” those etiologies needing medical evaluation and therapy as soon as possible in order to prevent potential sequelae, including oncologic etiologies, bone or neurological infections and other pathologies such as epiphysiolysis, Perthes disease, Guillain Barrè syndrome and non-accidental injuries [2,14].

We excluded from our analysis those patients with a previously established medical condition as the main cause of lameness.

### 2.2. Statistical Analysis 

Statistical analyses were conducted using STATA/IC 14.2 (2017). Normality was assessed using the Skewness/Kurtosis test. Data were presented as median values with an interquartile range (IQR), and direct comparisons were made with the Kruskal–Wallis test. Percentages were used to describe the categorical outcomes, and distributions of categorical data were compared with either Pearson’s χ^2^ test or Fisher’s exact test. A multinomial logistic regression was performed to detect those factors thought to be associated with specific etiologies and prognosis of limping patients; we chose patients’ age as the reference category. The inclusion of variables in the model was based on clinical plausibility and significant differences in the bivariate analysis. Adjusted odds ratio (OR) and 95% confidence intervals (95% CI) were used as measures of effect. Statistical significance was set at *p* < 0.05 for all tests.

This study adhered to the principles outlined in the Declaration of Helsinki and obtained approval from the Institutional Ethics Committee of Bambino Gesù Children’s Hospital, IRCCS (Date 15 May 2018; Number 1565_OPBG_2018).

## 3. Results

Over the 2-year study period, a total of 113,623 admissions were recorded in our PED.

Following a keyword search, 485 patients up to 16 years old were evaluated in the PED for non-traumatic limping during the study period. Their medical charts were manually reviewed, confirming their eligibility. The observed incidence was 4.27 cases per 1000 admissions.

The average age was 5.2 years, with a male predominance (62%). The majority fell into the children’s age group (3–10 years, 55.6%), while toddlers (<3 years) and adolescents (>10 years) constituted 25.2% and 19.2%, respectively. In 96.9% of the cases, a minor triage code was assigned, with “leg pain” being the primary reason for PED presentation (84.9%). The average time between symptom onset and PED access was 2 days (1–6). 

Eighty-nine patients (18.3%) reported at least one previous episode of limping. 

During clinical evaluation, 19.5% exhibited at least one red flag symptom; fever, fatigue and night pain were the most frequently reported (36.7%, 6.7% and 6.7%, respectively). Patients diagnosed with a crippling condition were more likely to present at least one red flag in the PED (36.7% of them).

Hip articulation was most commonly involved during examination (127 patients, 26.1%), followed by the knee (20 patients) and ankle (10 patients); many patients reported diffuse articular tenderness.

Differences were noted in the incidence of clinical manifestations and management based on patient age. Notably, an urgent triage tag was more frequently assigned to toddlers (7.5% of patients aged <3 years old, *p* = 0.02), who were also more likely to report a recent history of respiratory infection (27%, *p* = 0.02) and exhibited refusal to walk or weight-bear (38.5%, *p* = 0.001) or who had a limited articular range of motion (31.1%, *p* = 0.015). Conversely, they less frequently complained of limb pain (71.3%, *p* < 0.001) (Table 2). 

Fever in the age group of children was unusual compared to the other age groups (21.4%, *p* = 0.01).

Among our adolescent patients, we found a longer duration of symptoms before presenting to the PED (median of 5 days, IQR 2–20, *p* < 0.001) and a more recurrent history of previous medical evaluation for limping (36.5% *p* = 0.02). 

About half of our patients (44.7%) underwent blood tests in the PED, with a higher median CPR revealed for toddlers (1.2 mg/dL, IQR 0.3–4.5, *p* = 0.001).

Imaging was performed for 321 patients (66.1%). Articular ultrasound was the most used investigation in the whole cohort (203 patients, 41.8%) and especially in the age group of toddlers (72.1% of them, *p* < 0.001); it revealed pathologic findings (such as joint effusion or synovitis) in 138 of the cases (67.9%).

X-rays were performed to evaluate 45 patients (9.2%) and were a first choice for adolescents (32.2% of them, *p* < 0.001), and showed significant radiological findings in 27 cases (60%); interestingly, two cases of fracture were revealed.

Fifteen percent of patients (especially adolescents: 25.8% of them, *p* = 0.006) required a specialistic evaluation in the PED, the neurological type being significantly more frequent in toddlers (46.6%, *p* = 0.012).

Only 16.5% of patients were hospitalized, which happened more frequently for toddlers (23.7% of them, *p* = 0.018).

The final diagnoses made in our PED are described in Table 3.

TSH was the most frequent one (34%), followed by arthromyalgia and musculoskeletal disorders (32.5%), while crippling conditions were identified in 6.2% of cases. 

Both groups of toddlers and children showed TSH as the most frequent diagnosis made in the study period (respectively, 44.2% and 34.8% of the latter, *p* < 0.001), while arthromyalgia was more likely among adolescents (54.8%, *p* < 0.001). 

Interestingly, we found two cases of scurvy as the presumed cause of limping. 

We made a diagnosis of a crippling condition for 30 patients (detailed in Table 4).

### 3.1. Multivariable Analysis

We performed a multinomial logistic regression (reported in Table 5), exploring the different clinical manifestations and management of our patients related to their age group. 

#### 3.1.1. Adolescents Relative to Children (the Base Outcome)

Female adolescents were 56% less likely to have an ED presentation for limping than female children (OR: 0.44; 95% CI: 0.26 to 0.74; *p* = 0.002). The odds of a high-priority triage code increased by almost 7 times for adolescents compared to children (OR: 6.91; 95% CI: 1.88 to 25.49; *p* = 0.004). 

The likelihood of a history of any prior ED evaluation increased by 21% in adolescents than in children (OR: 1.21; 95% CI: 1.01 to 1.46; *p* = 0.042).

Adolescents were over 3 times more likely to report limb pain (OR: 3.44; 95% CI: 1.12 to 10.52; *p* = 0.030) and to present warm joints compared to children (OR: 3.02; 95% CI: 1.03 to 8.87; *p* = 0.04), while fever was 71% less likely to be found in their age group (OR: 0.29; 95% CI: 0.12 to 0.71; *p* = 0.007). 

#### 3.1.2. Toddlers Relative to Children (the Base Outcome)

Compared to the group of children, pain was 66% less likely to occur in toddlers (OR: 0.37; 95% CI: 0.20 to 0.67; *p* = 0.001), while the odds of showing refusal to walk or weight-bear were almost twice greater in their group (OR: 1.88; 95% CI: 1.11 to 3.17; *p* = 0.02). Painful passive motion of the lower limb was 47% less probable to occur in toddlers (OR: 0.53; 95% CI: 0.32 to 0.86; *p* = 0.01), while the limited range of motion was twice more likely to occur in their age group than the children’s one (OR: 1.96; 95% CI: 1.07 to 3.57; *p* = 0.03).

## 4. Discussion

To the best of our knowledge, this study constitutes a comprehensive pediatric case series on acute LC within a single pediatric ED. These incidences occur in less than 5 cases per every 1000 ED consultations, highlighting that this is not an uncommon condition in the pediatric emergency setting. 

However, the significance of these cases should not be understated, as they raise concerns for family members and pediatricians. The symptoms associated with acute LC may be indicative of an underlying and potentially severe cause, warranting careful attention and consideration. 

For the purposes of our study, we retrospectively reviewed the medical charts of 485 patients evaluated in our tertiary PED for non-traumatic limping during the study period and divided them into three groups of age (toddlers, children and adolescents). Most of them were male children (aged 3 to 10 years old) complaining of leg pain for less than a week.

There is currently scarce research about the management of limping patients in PED settings, along with significant variability among different centers.

In our cohort, a serious disease was diagnosed in a significant number of patients (6.2%, which is in line with the existing literature), and our finding that none of them had been assigned an urgent triage tag at the entrance highlights the difficulty in the diagnostic work-up in this population. 

We were unable to assess and verify the clinical, laboratory or instrumental predictors, mostly due to the unavailability of follow-up data within our cohort.

TSH was found to be the most frequent etiology in our cohort of patients, according to the literature data [18,19,20,21]; notably, it was diagnosed in large part in our toddlers’ age group, despite it being commonly imputed to older patients. 

The underlying reasons for this can be referred to the high use of hip ultrasound in our cohort, though it is not generally considered mandatory to make a diagnosis of TSH, increasing the chances to detect possible joint effusion and other signs of inflammation; in fact, in our center, we dispose of an Emergency radiology Department which is open 24 h every day of the year, and this can encourage our physicians to use non-invasive imaging such ultrasound in support of their clinical activity. However, it is important to underline that the differential diagnosis of TSH in children is a crucial point, especially in younger children and especially under 2 years of age. In emergency settings, we primarily try to exclude infectious conditions, such as septic arthritis. We consider continuity of care essential in the event of discharge from the Emergency Department. In fact, we believe that close follow-up is mandatory after discharge from the emergency room directly in our clinics if the child is not hospitalized for further checks.

Moreover, clinical manifestations can be mild and nonspecific in limping toddlers, whose pain in lower limbs can be hard to estimate; moreover, for their inability to describe anamnestic events, a possible misdiagnosis with tibial torus fractures has to be taken into account [22]. In this perspective, ultrasound can be helpful for differential diagnosis [23].

The data extracted from our analysis suggest that a larger use of ultrasound could also be indicated for LC since they are less likely to present typical clinical manifestations than toddlers, such as a limited articular range of motion and weight-bearing.

Ultrasound has been considered a primary imaging tool for a child with a non-traumatic limp, being more sensitive in detecting joint effusion with respect to X-rays. We believe that if, in addition to the limping the patient feels pain in the affected skeletal segment upon physical examination, we perform an X-ray of the affected segment in the PED. In fact, in patients with a suspected fracture, slipped capital femoral epiphysis or Perthes disease, an X-ray is the first choice for imaging investigation. Finally, it should be remembered that MRI is an extremely useful excellent tool in the early diagnosis of various potentially severe conditions as occurs in the case of diagnosis of osteomyelitis. [16]. 

Arthromyalgia was found in a great number of adolescents, especially girls. These data are in line with the literature and can be at least partially explained by the role of continuous osteoarticular stress linked to sports activities at this age, also due to the possibility of minor traumas or injuries possibly not reported by patients [24,25]; furthermore, sometimes adolescents are known to show a tendency to somatization [26]. 

We reported many rheumatologic disorders as the final diagnosis; it is to say that in our study, we included heterogeneous conditions under this term, and this is because those patients were meant to be sent to a rheumatologic specialist to continue their ambulatory diagnostic workout and follow-up. Again, as a third-level hospital, patients with suspected specialistic diseases are often sent to our attention [27], and there is probably a tendency to remit the PED diagnostic workout to specialistic departments.

Notably, in two cases, we made a diagnosis of scurvy that seemed to justify lameness; this could serve as a warning when considering rare causes of limping and not just in the case of patients coming from low socioeconomic contexts [28].

Our data suggest paying particular attention to limping toddlers and searching for the presence of red flags, but their absence should not exclude the possibility of severe underlying conditions (they were present in 36.7% of our patients with serious etiologies). Specifically, in our cohort, we found fever (the most frequently reported red flag), night pain and fatigue to be the warning symptoms significantly associated with severe conditions.

The most described symptom among our patients was pain at passive motion of lower limbs, also as a consequence of the high number of toddlers in our population since obviously they cannot complain of pain during active mobilization.

Adolescent patients accounted for a small proportion of our population, and interestingly, they were assigned higher priority triage codes; this can be due to their capability to report and describe pain, which, in fact, they were more likely to present than children (OR 3.44). It is crucial to underline that older children must be evaluated by specialists early in order to have a proper diagnosis.

Also, evaluating the presence of recurrent limping can be helpful in distinguishing medical conditions that are potentially deserving of second-level exams. In fact, 26.6% of patients with severe conditions had a history of previous PED access for lameness, and this highlights the importance of establishing an appropriate follow-up, especially for those cases of limping that are not investigated with second-level exams in a PED.

In case of diagnostic doubt and when accessible, specialist consultancy can reduce the risk of misdiagnosis (60% of our patients with severe conditions had been evaluated by specialists). 

Finally, our study confirmed the existing data about the percentage of hospitalizations for limping patients in PED settings.

The main limitations of this study are its retrospective design and the lack of follow-up data within our cohort of patients (except for the hospitalized ones); this is particularly important, given the lack of predictor tools in the PED and the unavailability of all necessary exams and investigations in this setting.

## 5. Conclusions

While occurring in less than five cases per 1000 PED consultations, LC presents a challenging dilemma in the acute setting.

Our study aimed to search for clinical and instrumental correlations (through a multinomial logistic regression) in the diagnostic work-up of patients presenting to a tertiary PED for lameness and to identify possible management strategies based on age.

Our data suggest that considering patients’ age can help physicians steer the diagnostic work-up for limping patients. 

In our cohort, a serious disease was diagnosed in a significant number of patients (6.2%, which is in line with the existing literature). Red flags (especially fever, night pain and asthenia) must be looked for since they are associated with serious causes of lameness.

TSH remains a major cause of limping in pediatrics; our analysis shows a higher incidence in toddlers than currently reported in the literature (probably due to the large use of ultrasound in our PED), but prospective studies assessing proper indications for imaging in limping toddlers for the search of TSH are needed.

Large prospective studies considering clinical data, laboratory and radiologic investigations and appropriate follow-ups are needed in order to validate an evidence-based approach for the management of non-traumatic limping in PEDs and to assess the epidemiology of final diagnoses with more accuracy.

## Figures and Tables

**Table 1:** Age-related most common diseases in pediatric patients presenting with atraumatic limp.

Toddlers (<3 Years)	Children (3–10 Years)	Adolescents (>10 Years)
Transient synovitis	Transient synovitis	Tumors
Reactive arthritis	Tumors	Septic arthritis
Septic arthritis	Septic arthritis	Osteomyelitis
Osteomyelitis	Osteomyelitis	Slipped capital femoral epiphysis
Painful gait	Osteochondritis dissecans	Juvenile idiopathic arthritis
Congenital limb deficiencies	Juvenile idiopathic arthritis	Apophyseal avulsions
Developmental dysplasia of the hip	Osteochondroses	Overuse syndrome
Metabolic disease	(Sever and Kohler disease)	Osteochondroses
Neuromuscular abnormalities	CRMO	(Osgood-Schlatter, van Neck–Odelberg and Sever disease)
Tumors	Malformative disease	CRMO
	Metabolic disease	Legg-Calvé-Perthes
	Legg-Calvé-Perthes	Metabolic disease
	Discoid meniscus	

CRMO = chronic recurrent multifocal osteomyelitis.

**Table 2:** The ED presentation of limping children: assessment of demographic, clinical and anamnestic characteristics in relation to age groups.

Characteristics	Total Population n = 485	Toddlers (<3 Years) n = 122 (25.2%)	Children (3–10 Years) n = 270 (55.6%)	Adolescents (>10 Years) n = 93 (19.2%)	*p*-Value
Sex, n (%)					**0.002**
Female	184 (37.94)	51 (41.80)	85 (31.48)	48 (51.61)	
Male	301 (37.94)	71 (58.20)	185 (68.52)	45 (48.39)	
Priority of ED visits, n (%)					**0.02**
Immediate-Urgent	15 (3.09)	7 (7.53)	1 (0.82)	7 (2.59)	
Not urgent-Delayed	470 (96.91)	121 (99.18)	263 (97.41)	86 (92.47)	
Timing of onset of symptoms at ED presentation (days), median (IQR)	2 (1–6)	1 (1–4)	1 (1–5)	5 (2–20)	**<0.001**
History of previous limping, n (%)	89 (18.35)	16 (13.11)	53 (19.63)	20 (21.51)	0.2
History of any prior ED evaluation, n (%)	131 (27.01)	24 (19.67)	73 (27.04)	24 (36.56)	**0.022**
Preceded by respiratory tract infection, n (%)	117 (24.12)	33 (27.05)	72 (26.67)	12 (12.90)	**0.02**
Pain, n (%)	412 (84.92)	87 (71.31)	236 (87.41)	89 (95.70)	**<0.001**
Pain at night, n (%)	19 (3.23)	4 (3.28)	12 (4.44)	3 (3.23)	0.8
Fever, n (%)	95 (19.59)	29 (23.77)	58 (21.48)	8 (54.75)	**0.01**
Sweating at night, n (%)	2 (0.41)	0	2 (0.74)	0	1
Weakness, n (%)	12 (82.47)	2 (1.64)	9 (3.33)	1 (1.08)	0.52
Headache, n (%)	3 (0.74)	0	2 (0.74)	1 (1.08)	0.8
Meningeal irritation, n (%)	2 (0.41)	0	2 (0.74)	0	1
Weight loss, n (%)	3 (0.62)	0	3(1.11)	0	0.59
Inability to bear weight on lower limb, n (%)	127 (26.19)	47 (38.52)	65 (24.07)	15 (16.13)	**0.001**
Pain with passive motion of lower limb, n (%)	296 (61.03)	60 (49.18)	177 (65.56)	59 (63.44)	**0.008**
Swelling of the joint, n (%)	56 (11.55)	20 (16.39)	25 (9.26)	11 (11.83)	0.12
Redness joint, n (%)	17 (3.65)	5 (4.10)	6 (2.22)	6 (6.45)	0.15
Warm joint, n (%)	31(6.39)	10 (8.20)	11 (4.07)	10 (10.75)	0.05
Limited range of motion, n (%)	106 (21.86)	38 (31.15)	49 (18.15)	19 (20.43)	**0.015**
Skin rash, n (%)	14 (2.89)	4 (3.28)	8 (2.96)	2 (2.15)	0.94
Myalgia, n (%)	36 (7.42)	3 (2.46)	25 (9.26)	8 (8.60)	**0.035**

**Table 3:** The description of the final diagnosis of limping relative to the age classification.

Final Diagnosis	Total Population n = 485	Toddlers (<3 Years) n = 122 (25.2%)	Children (3–10 Years) n = 270 (55.6%)	Adolescents (>10 Years) n = 93 (19.2%)	*p*-Value
Transient synovitis, n (%)	165 (34.02)	54 (44.26)	94 (34.81)	17 (18.28)	**<0.001**
Arthromyalgia, n (%)	158 (32.58)	26 (21.31)	81 (30.00)	51 (54.84)	**<0.001**
Rheumatological disorder, n (%)	70 (14.43)	22 (18.03)	42 (15.56)	6 (6.45)	**0.04**
Infectious disease, n (%)	50 (10.31)	9 (7.38)	34 (12.59)	7 (12.59)	0.18
Musculoskeletal disorder, n (%)	20 (4.12)	3 (2.46)	8 (2.96)	9 (9.68)	**0.02**
Oncological disease, n (%)	6 (1.24)	3 (2.46)	3 (1.11)	0	0.26
Neurological disorder, n (%)	5 (1.03)	2 (1.64)	2 (0.74)	1 (1.08)	0.71
Neuropsychiatric disorder, n (%)	5 (1.03)	1 (0.82)	2 (0.74)	2 (2.15)	0.5
Metabolic-Nutritional Abnormality, n (%)	2 (0.41)	0	2 (0.74)	0	0.45
Crippling conditions, n (%)	30 (6.19)	13 (10.66)	11 (4.07)	6 (6.45)	**0.043**

**Table 4:** Description of the crippling conditions found in our cohort.

Crippling Conditions	Number of Patients
Legg-Calve-Perthes disease	6
Septic arthritis	6
Leukemia	4
Slipped Capital Femoral Epiphysis	3
Osteomyelitis	2
Spondylodiscitis	2
Guillain-Barré syndrome	2
Cerebellitis	2
Sacrococcygeal abscess	2
Neuroblastoma	1

**Table 5:** Multinomial logistic regression analyses determining the factors influencing the presentation of limping at ED in relation to the age groups. The base outcome was the child’s age group.

Age Group	OR	Std. Err.	Z	*p* > |z|	95% CI	
**Adolescent vs. children**						
**Sex** Female vs. male	0.44	0.12	−3.08	**0.002**	0.26	0.74
**Priority of ED visits** Immediate-Urgent versus Not urgent-Delayed	6.91	4.60	2.9	**0.004**	1.88	25.49
**Timing of onset of symptoms (days)**	1.003	0.001	2.72	**0.007**	1.00	1.005
**History of any prior ED evaluations**	1.21	0.11	2.04	**0.042**	1.01	1.46
**Preceded by respiratory tract infection**	0.52	0.19	−1.78	0.076	0.26	1.07
**Pain**	3.44	1.96	2.16	**0.030**	1.12	10.52
**Fever**	0.29	0.13	−2.7	**0.007**	0.12	0.71
**Shortening or avoiding of the** **stance to unload the lower limb**	0.56	0.21	−1.58	0.11	0.27	1.15
**Painful passive motion of the lower limb**	0.76	0.22	−0.97	0.33	0.43	1.33
**Warm joint**	3.02	1.66	2.01	**0.04**	1.03	8.87
**Limited range of motion**	1.23	0.45	0.57	0.57	0.60	2.52
**Myalgia**	1.58	0.77	0.95	0.34	0.61	4.10
**Constant**	0.20	0.11	−2.8	0.01	0.06	0.62
**Toddlers vs. children**						
**Sex** Female vs. male	0.69	0.17	−1.56	0.119	0.43	1.10
**Priority of ED visits** Emergency-Urgent versus Not urgent-Delayed	0.32	0.36	−1	0.315	0.04	2.92
**Time of onset symptoms**	0.995	0.004	−1.28	0.201	0.987	1.003
**History of any prior ED evaluations**	0.90	0.11	−0.87	0.387	0.71	1.14
**Preceded by respiratory tract infection**	1.06	0.29	0.21	0.837	0.62	1.80
**Pain**	0.37	0.11	−3.3	**0.001**	0.20	0.67
**Fever**	1.12	0.34	0.37	0.711	0.62	2.02
**Shortening or avoiding of the** **stance to unload the lower limb**	1.88	0.50	2.37	**0.02**	1.11	3.17
**Painful passive motion of the lower limb**	0.53	0.13	−2.56	**0.01**	0.32	0.86
**Warm joint**	2.01	1.02	1.38	0.17	0.75	5.41
**Limited range of motion**	1.96	0.60	2.2	**0.03**	1.07	3.57
**Myalgia**	0.30	0.19	−1.87	0.06	0.08	1.06
**Constant**	1.41	0.45	1.08	0.28	0.76	2.63

OR = Odds ratio. These are the relative risk ratios for the multinomial logit model. They can be obtained by exponentiating the multinomial logit coefficients, e^coef^. Std. Err = standard errors associated with the coefficients. z and *p* > |z|—These columns provide the z-value and 2-tailed *p*-value used in testing the null hypothesis that the coefficient (parameter) is 0. 95%. CI = This is the confidence interval for the relative risk ratio, given that the other predictors are in the model.

## Data Availability

The data presented in this study are available on request from the corresponding author. The data are not publicly available due to privacy protection.

## Abbreviations

LC	Limping child
PED	Pediatric Emergency Departments
TSH	Transient synovitis of the hip

## References

[1] Fischer S.U., Beattie T.F. (1999). The limping child: Epidemiology, assessment and outcome. J. Bone Jt. Surg. Br..

[2] Lázaro Carreño M.I., Fraile Currius R., García Clemente A. (2018). Non traumatic limping in Paediatric Emergencies: Epidemiology, evaluation and results. Rev. Esp. Cir. Ortop. Traumatol..

[3] Herman M.J., Martinek M. (2015). The limping child. Pediatr. Rev..

[4] Sen E.S., Clarke S.L., Ramanan A.V. (2014). The child with joint pain in primary care. Best. Pract. Res. Clin. Rheumatol..

[5] Bartoloni A., Aparisi Gómez M.P., Cirillo M., Allen G., Battista G., Guglielmi G., Tomà P., Bazzocchi A. (2018). Imaging of the limping child. Eur. J. Radiol..

[6] Whitelaw C.C., Varacallo M. (2022). Transient Synovitis. StatPearls [Internet].

[7] Kocher M.S., Zurakowski D., Kasser J.R. (1999). Differentiating between septic arthritis and transient synovitis of the hip in children: An evidence-based clinical prediction algorithm. J. Bone Jt. Surg. Am..

[8] Barkin R.M., Barkin S.Z., Barkin A.Z. (2000). The limping child. J. Emerg. Med..

[9] Luhmann S.J., Jones A., Schootman M., Gordon J.E., Schoenecker P.L., Luhmann J.D. (2004). Differentiation between septic arthritis and transient synovitis of the hip in children with clinical prediction algorithms. J. Bone Jt. Surg. Am..

[10] Delaney R.A., Lenehan B., O’sullivan L., McGuinness A.J., Street J.T. (2007). The limping child: An algorithm to outrule musculoskeletal sepsis. Ir. J. Med. Sci..

[11] Reed L., Baskett A., Watkins N. (2009). Managing children with acute non-traumatic limp: The utility of clinical findings, laboratory inflammatory markers and X-rays. Emerg. Med. Australas..

[12] McCanny P.J., McCoy S., Grant T., Walsh S., O’Sullivan R. (2013). Implementation of an evidence based guideline reduces blood tests and length of stay for the limping child in a paediatric emergency department. Emerg. Med. J..

[13] Naranje S., Kelly D.M., Sawyer J.R. (2015). A Systematic Approach to the Evaluation of a Limping Child. Am. Fam. Physician.

[14] Adamson J., Waterfield T. (2020). Fifteen-minute consultation: The limping child. Arch. Dis. Child. Educ. Pract. Ed..

[15] Payares-Lizano M. (2020). The Limping Child. Pediatr. Clin. N. Am..

[16] Tu J., Haines M., Gowdie P., Craig S. (2023). Paediatric acute non-traumatic limp presenting to the emergency department: A retrospective observational study. Emerg. Med. J..

[17] Conferenza Permanente per i Rapporti tra lo Stato le Regioni e le Province Autonome di Trento e Bolzano. Accordo tra il Ministro Della Salute, le Regioni e le Province Autonome sul Documento di Linee-Guida sul Sistema di Emergenza Sanitaria Concernente: “Triage Intraospedaliero (Valutazione Gravità all’Ingresso) e Chirurgia della Mano e Microchirurgia nel Sistema dell’Emergenza—Urgenza Sanitaria”. Gazzetta Ufficiale Serie Generale n.285. https://www.gazzettaufficiale.it/eli/id/2001/12/07/01A12203/sg.

[18] Hart J.J. (1996). Transient synovitis of the hip in children. Am. Fam. Physician.

[19] Nouri A., Walmsley D., Pruszczynski B., Synder M. (2014). Transient synovitis of the hip: A comprehensive review. J. Pediatr. Orthop. B.

[20] Lipshaw M.J., Walsh P.S. (2022). Transient synovitis of the hip: Current practice and risk of misdiagnosis. Am. J. Emerg. Med..

[21] Morancie N.A., Helton M.R. (2023). Evaluating the Child with a Limp. Am. Fam. Physician.

[22] Seyahi A., Uludag S., Altıntaş B., Demirhan M. (2011). Tibial torus and toddler’s fractures misdiagnosed as transient synovitis: A case series. J. Med. Case Rep..

[23] Martinoli C., Garello I., Marchetti A., Palmieri F., Altafini L., Valle M., Tagliafico A. (2012). Hip ultrasound. Eur. J. Radiol..

[24] Beck B., Drysdale L. (2021). Risk Factors, Diagnosis and Management of Bone Stress Injuries in Adolescent Athletes: A Narrative Review. Sports.

[25] Frush T.J., Lindenfeld T.N. (2009). Peri-epiphyseal and Overuse Injuries in Adolescent Athletes. Sports Health.

[26] Kozlowska K. (2013). Functional somatic symptoms in childhood and adolescence. Curr. Opin. Psychiatry.

[27] Pollack M.M., Alexander S.R., Clarke N., Ruttimann U.E., Tesselaar H.M., Bachulis A.C. (1991). Improved outcomes from tertiary center pediatric intensive care: A statewide comparison of tertiary and nontertiary care facilities. Crit. Care Med..

[28] Ceglie G., Macchiarulo G., Marchili M.R., Marchesi A., Rotondi Aufiero L., Di Camillo C., Villani A. (2019). Scurvy: Still a threat in the well-fed first world?. Arch. Dis. Child..

